# Approaches to Tailoring Between-Session Mental Health Therapy Activities

**DOI:** 10.1145/3613904.3642856

**Published:** 2024-05-11

**Authors:** Bruna Oewel, Patricia A. Areán, Elena Agapie

**Affiliations:** University of California, Irvine Irvine, USA; University of Washington Seattle, USA; University of California, Irvine Irvine, USA

**Keywords:** mental health, goal setting, behavior change, personalization, tailoring

## Abstract

Mental health activities conducted by patients between therapy sessions (or “therapy homework”) are a component of addressing anxiety and depression. However, to be effective, therapy homework must be tailored to the client’s needs to address the numerous barriers they encounter in everyday life. In this study, we analyze how therapists and clients tailor therapy homework to their client’s needs. We interviewed 13 therapists and 14 clients about their experiences tailoring and engaging in therapy homework. We identify criteria for tailoring homework, such as client skills, discomfort, and external barriers. We present how homework gets adapted, such as through changes in difficulty or by identifying alternatives. We discuss how technologies can better use client information for personalizing mental health interventions, such as adapting to client barriers, adjusting homework to these barriers, and creating a safer environment to support discomfort.

## 1 INTRODUCTION

Mental health is a serious public health issue. Depression and anxiety affect over 16 million American adults each year [2]. Of all American adults, about one in five received treatment for mental health issues in 2021 [3, 117]. Mental health can be managed through mental health therapy [12, 15, 41], which includes between-session therapy activities, also known as “therapy homework”. Therapy homework involves personalized goals and activity plans that enable clients to practice and strengthen therapeutic skills, and engage in behaviors that promote mental health [25, 65, 68]. Between-session therapy activities have been effective at alleviating symptoms of mental health problems, such as depression and anxiety [31, 68].

Therapy homework should be aligned with the client’s needs to be effective [119]. To improve alignment, therapists work closely with their clients, often over multiple sessions, to identify their goals and suggest between-session activities for the client [12, 15, 40, 41, 55]. They employ their client’s values, long-term goals, underlying problems, and social relationships to tailor activities [7, 15, 33, 55, 119]. Without good tailoring, clients might encounter barriers such as low motivation to change or lack of understanding the relevance of therapy homework. They might also struggle to devote sufficient time to homework because they lack time, or daily priorities come in the way [24, 25, 47]. Understanding therapists’ practices can help inform how technology can better incorporate tailored interventions for mental health.

Tailoring behavioral activities can be difficult to achieve in the design of technology aimed at setting goals [7], providing behavioral recommendations and behavioral plans [10, 33], and delivering motivational messages [20, 69]. Tailoring of technologies is limited when they do not account for the contexts people experience — such as location (e.g., home vs. work) [93] and timing for engagement in desired behaviors [89, 93] — people’s values [69], and how people engage in behaviors (e.g., being “on the go” and eating unhealthy food) [33]. Adaptive interventions, which personalize the type, content, or timing of behavioral interventions to the user context, can personalize behavioral support [20, 84, 127], yet they do not account for people’s ever-changing lives which can pose obstacles to people engaging in the intended interventions [112, 127].

Many lessons about how behavioral interventions are tailored can be drawn from behavioral counseling. Research in HCI has surfaced approaches that providers use to tailor support for their clients to surmount mental health challenges. Health providers, behavioral counselors, and coaches tailor client goals or action plans [21, 33, 97, 98, 103] through understanding the client’s needs, preferences, values, and life context, and matching them to the client’s goals [21, 98]. Although we have some understanding of the information necessary for tailoring, it is unclear how clients and providers adjust behavioral interventions to support client needs. Mental health is a particularly challenging domain for personalizing behavioral interventions to support people’s goals because the goals people set are holistic and span multiple aspects of their life (e.g. exercise, diet, relationships, and symptom management) [7]. People also might manage multiple such goals [7], prioritize different goals at different times [7], and experience a wide range of mental health symptoms (e.g., fatigue or sadness) [6, 70] that can affect people’s motivation to work towards their goals [45]. It is unclear which aspects of a person’s individual circumstance and life are most important to tailoring mental health support, and how providers decide how to adjust activities based on client needs.

In this paper, we investigate how mental health therapists and clients tailor between-session activities, or therapy homework, to the client’s needs. To understand common challenges clients and therapists experience when setting and implementing homework, and how they adjust homework to fit their clients’ needs, we interviewed 13 mental health therapists and 14 clients. We identify techniques that clients and therapists use to tailor therapy homework and the challenges clients experience in doing homework in their everyday lives. We propose design implications about how technology could better support clients and therapists in this process. Our work contributes:

- Understanding of the challenges that clients experience while engaging in mental health activities: external barriers, discomfort, and mismatches of between-session therapy activities and client skill.
- Understanding of how mental health therapists and clients tailor between-session therapy activities to the client’s needs by adapting the difficulty of activities, identifying alternative activities in the face of barriers, and supporting engagement with uncomfortable mental health activities.
- Implications for how technology can better support tailoring of client mental health activities and engagement by tailoring mental health interventions to client skills, locations, and availability. We propose approaches to adjusting mental health activities, and designing experiences to address discomfort through technology.

## 2 RELATED WORK

Behavioral activities can support people’s pursuit of goals. We describe the importance of understanding the tailoring practices of health counselors and the approaches they use to tailor behavioral interventions. We then describe how tailoring behavioral support is implemented into digital tools within and outside mental health, and the limitations of such tailoring.

### 2.1 Provider-driven tailoring of behavioral activities

Evidence-based behavioral interventions and treatments used by clinicians, health coaches, and counselors often involve using a client-centric approach: recommendations are tailored to the needs of clients, which helps clients engage with health behaviors [12, 41, 46, 61, 96, 123]. The techniques used by professionals to support clients implementing health behaviors involve close interaction with the client, to understand and assess their needs, identify an appropriate approach for supporting the health behavior, and tailor support to the client’s needs and situation [46, 54, 123]. Professionals tailor their recommendations based on client characteristics including internal factors—such as client preferences, motivations, and values—and external factors—such as the client’s life context [21, 98]. For example, dietitians assess client needs to tailor educational content and goals to the client’s preferences, food constraints, experiences, context, and unique challenges [33, 75, 96, 98]. Personal trainers similarly help clients create exercise programs that are tailored to the level of client’s fitness level, goals, preferences, and constraints [46]. Smoking cessation counselors adapt their interventions to client preferences or availability of social support [21].

#### Tailoring behavioral activities in mental health: therapy homework.

Mental health treatments such as Behavioral Activation, Cognitive Behavioral Therapy, and Cognitive Processing Therapy [31, 68] encourage setting goals and planning activities for the client to do between sessions. Those activities are referred to as “homework” in therapy practice, and they are essential to achieving long-term therapy goals [65]. Therapy homework encompasses activities that can help the client practice therapeutic skills in real-world situations and environments [25, 66]. Examples of activities set during therapy homework include behavioral skills training—such as social activities, exercise, or physical activities—and self-care or reflective activities [65]. Therapists strive to tailor therapy homework in their practice [64].

Tailoring in mental health therapy emphasizes adjusting homework to client needs. That is, they should incorporate the client’s values, problems, and socio-cultural context [7, 15, 41, 55, 119]. The homework should be achievable between therapy sessions [119] and align with the coping strategies of the client [35]. Therapy homework should include, when, where, how often, and how long a strategy should take [64]. Even though such best practices exist, how therapists adjust therapy homework in practice is not well documented. In this research we investigate client needs for adapting homework to their situations, and practices for how clients and therapists identify such adjustments.

### 2.2 Technology tailoring for behavioral activities

#### 2.2.1 Tailoring criteria and approaches for behavioral technologies.

Despite allowing for adaptation, technology still offers only limited support for tailoring behavioral activities. To describe tailoring in current technologies, we draw from literature on just-in-time adaptive interventions (JITAIs). They systematically describe dimensions for adapting interventions that capture user context and can be useful for characterizing different aspects of tailoring an intervention [60, 84]. Based on Nahum-Shani et al., to support a person’s long-term goal [84], system designers should use *tailoring variables*—information about the individual that is used to customize when and how to intervene. Based on user context, a personalized system should consider which *intervention option* to deliver at a *decision point* through a *decision rule*. *Decision points* are points when an intervention decision can be made. *Decision rules* are rules that help decide which intervention a technological system can offer, for whom, and when. *Intervention options* are possible treatments that might be used at any given decision point. When investigating the design of just-in-time interventions, we found systems only incorporate tailoring variables to a limited extent [60]. They included variables such as location [22, 122], activity (e.g. watching TV) [44], alarms [122], number of calendar events [56], time of the day [44, 122], and past responses of users (such as feedback or personal preferences) [22, 107].

HCI research has found that contextual aspects of managing health behaviors might not be incorporated into technology, or are implemented in limited ways. In HCI, aspects of context such as location, time, social environment, or current behaviors can be part of tailoring variables that are used to adapt interventions, and decision rules about which intervention to deliver to users. Different locations (e.g., home vs. work) [93] and times of day might pose opportunities to engage in desired behaviors [89]. Different periods of time might be characterized by different user behaviors. Students who are home during summer manage diabetes differently than when they are in school [93], and people managing physical rehabilitation at home need to negotiate space with family [13]. Certain times of day might be more opportune for engaging in behaviors like walking [89]. When activities are routinized, they can be easier to engage in than when they are new or occur in unexpected situations [27, 85]. The situations individuals are involved in influence how they engage or disengage with healthy behaviors (e.g., eating a snack might be hard to resist when one is studying for a long period) [125]. Technologies lack adaptability to changes in people’s skills, barriers, and goals [7, 102].

Technologies increasingly support users in tailored or customized ways when it comes to *intervention options* for behavioral goals. For example, to adapt technology to health goals, technologies prompt the user to set a “step goal”—a minimum number of steps and the minimum number of days per week they would achieve it [83]. They also can prompt users to set backup or alternate goals [32, 82], margins for their goals [8, 59], difficulty of the goals based on progress [73, 83], and specific actionable goals through planning [9, 10]. Technology can also support people by adjusting the properties of the behaviors they intend to pursue. These properties include frequency of engaging in a behavior (like exercise or smoking) [9, 54], where and with whom an individual might do an activity [9], and the amount of effort an activity requires [56]. Individualized support can help a person adapt activities to achieve their goals, such as what exercises to do or what foods to eat, by taking their preferences into account [9, 33]. Technology can help people identify what behaviors work best for them, such as by identifying food triggers or effective sleep strategies [62, 63, 72].

#### 2.2.2 Tailoring criteria and approaches in mental health technologies.

Many mental health technologies can be used by individuals to increase access to mental health resources when they aren’t engaged in therapy [94, 95], or to complement therapy by practicing skills learned in therapy [104, 114]. Technologies include a range of functionalities that are sometimes part of a single app [4] or of a suite of apps [79]. Technologies incorporate a range of interventions including: psycho-educational content, goal setting, cognitive reframing, emotion regulation, tracking behavior, and sleep hygiene [4, 79, 105].

Research in digital mental health shows the importance of different tailoring variables in the delivery of mental health interventions. For example, different times of day are correlated with different symptoms of depression and anxiety [50, 116]. Systems have started using contextual data such as the user’s location [17, 48, 58], time [17, 58], motion (e.g. walking, running) [30, 58], sleep [17, 80], social context [17, 80], and the location’s current weather [58] to understand user behavior and mental states, or to customize interventions. Technologies use phone sensors to leverage information such as location, light, and recent calls to predict people’s mood [26, 88, 120, 126]. Studies have used unobtrusive smartphone sensor data collection of contextual data (e.g., sleep duration, motion, or physical activity) to model changes in mental health outcomes such as daily stress, depression, and loneliness [17, 26, 126].

Despite context being used in predicting mental health, it is unclear how context can be used for tailoring recommendations [20, 57]. For example, text messages are adapted based on the user’s changes in location [48], proximity to others [48], screen status [88], and proximity to home or work [88]. However, tailoring variables needed in mental health interventions can be uniquely complex, and requires data difficult to detect with phone sensors. Even though research shows the potential of leveraging tailoring variables for personalization, current consumer-facing technologies overwhelmingly focus on the time of executing the goal, reflecting on the goal, and customizing the interface [14, 111]. Decision rules for when to tailor an intervention could involve engaging users differently at different moments, particularly by avoiding moments of negativity [70], changes in mental health symptoms [39, 70, 87], and instability [70]. Such tailoring can help individuals break down goals when they feel less motivated, so that they can engage in mental health activities later [70]. Researchers point to a need to better understand how to tailor digital mental health interventions by incorporating user needs and context into technology [20, 70, 120]. For example, research is necessary to understand how interventions support user engagement in mental health activities by adapting them to factors such as busy times of day, social situations, and different affective moods [20].

In this research, we propose understanding the challenges clients experience in engaging in homework, and the tailoring practices of mental health therapists and clients to address those challenges. The range of activities supported in mental health apps (e.g., cognitive restructuring, journaling, and behavior activation) are part of therapy homework. Understanding how clients and therapists tailor homework to the client’s needs can give us insights into how technology can be designed to better tailor mental health activities to the individual.

## 3 METHODS

We conducted interviews with 13 mental health therapists and 14 patients to understand the challenges they faced while identifying and implementing therapy activities intended to be used outside of therapy sessions, and what potential solutions would address those challenges. This study fits the “exempt” category of IRB requirements at our institution, based on consultations with the staff of our IRB board. We assessed that the study was unlikely to pose risks such as criminal or civil liability, or impact on financial standing and employability. The interviews focused on the therapy practice of designing and engaging in homework activities, and understanding how this practice took place in therapy sessions and client’s everyday experiences. Our interviews did not include questions about the mental health conditions of participants. We took additional measures to ensure that participants could maintain anonymity by not requesting the client’s real names. We encouraged the clients to not disclose any information they were not comfortable sharing. We asked therapists to never disclose client names and information, only describe how they design homework, and not refer to any specific clients. We audio-recorded and transcribed each participant’s interview. Identifiable information was not captured in the recordings.

### 3.1 Participant Recruitment

#### Therapist participants.

We interviewed 13 mental health therapists, who we recruited through the mailing list of the Association for Behavioral and Cognitive Therapies (11) and convenience sampling (2). We received responses from 19 eligible participants, and interviewed 13 therapists. Participants were required to be (1) over the age of 18, (2) working or living in the US, and (3) and practicing a therapy that includes goal-setting and/or therapy homework. The interviews were conducted over Zoom and lasted 60–80 minutes. We compensated participants with a $40 gift card.

Four therapist participants were PhD candidates in Clinical Psychology but had been delivering therapy for 3 to 4 years; 7 participants had a PhD in Clinical Psychology; and 2 were Licensed Clinical Social Workers (LCSW). Participants met the following inclusion criteria: they had 3–5 years of experience (5), 6–9 years of experience (7), and over 20 years of experience (1). The types of therapy they practiced were Cognitive Behavioral Therapy (CBT), Dialectical Behavioral Therapy (DBT), Acceptance and Commitment Therapy (ACT), Cognitive Processing Therapy (CPT), Problem Solving Therapy (PST), and Behavioral Activation (BA). Participants practiced in 8 different states in the USA. Participants reported their gender as man (1), woman (9), and did not specify (3). Participant ages ranged from 25–34 (10), 35–44 (2), and 45–54 (1). Participants self-identified as Caucasian/white and Not Hispanic/Latino(a) (10), Middle Eastern (1), or did not specify (2).

#### Client participants.

We recruited 14 therapy clients through posts on social media (Reddit: r/depression and r/anxiety, Twitter, Instagram, Facebook), a mental health newsletter (Mental Health America), and Craigslist. Participants met the following inclusion criteria: they were (1) over the age of 18, (2) living in the US, (3) seeing a therapist and were practicing goal-setting and/or homework during therapy sessions, (4) self-identified as depressed or anxious, but did not have a severe mental illness condition, and (5) were seeing a therapist regularly at the time of the interview or up to two weeks prior to the interview. We excluded participants who had severe mental illness conditions because the therapy they might be receiving could involve techniques in addition to behavioral therapies, which would modify the treatment they receive and how they might engage with therapy homework [65]. A total of 39 survey respondents were eligible to participate, and 18 responded to scheduling an interview. We excluded 2 participants because they had severe mental health conditions that they disclosed during the interview, and for which treatment might be different than the rest of our sample. We excluded 2 participants due to not having homework during therapy. The interviews were conducted over Zoom and lasted 60–80 minutes. Participants were compensated with a $30 gift card.

Client participants reported their gender as man (3) and woman (11). Participant ages ranged from 25–34 (5), 35–44 (6), 45–54 (2), and over 60 (1). Participants self-identified as Caucasian/White (9) or Asian or South Asian (5). One person self-identified as Hispanic/Latino(a). Participants were attending therapy sessions weekly (6), biweekly (4), or monthly (3). Participants reported using the following therapies during therapy sessions: CBT (6), DBT (1), integrative therapy (1), or did not know or specify (6).

The participant demographic was skewed toward women, as only 8% of the therapists and 21% of clients we interviewed self-identified as men. Although the number of men participants was low, it is consistent with demographic trends in the gender of mental health therapists and clients seeking care; studies have shown that almost twice as many women were diagnosed with depression or received mental health treatment than men [71, 108]. For example, in 2019, 24.7% of American women received care compared to 13.4% of men [117]. Similarly, mental health therapy providers are predominantly women, a gender bias that can be traced back to their education. Seventy-five percent of psychology students in the US were women in 2014 [34], which is similar to the statistic that 74% of psychologists in the US were women in 2021 [5].

### 3.2 Data Collection

We conducted semi-structured interviews accompanied by activities, which prompted discussion about the challenges participants encountered in goal setting, using therapy homework during therapy sessions, and the role of technologies in this process. We used a digital collaborative whiteboard (Miro.com) to guide the interview activities. First, both therapists and clients discussed the main challenges they encountered in goal setting and homework, which they also sent us upon recruitment by email. We used digital sticky notes on the digital whiteboard to facilitate conversation about the participant’s challenges. We included sticky notes with common challenges of therapy goal setting and homework identified by previous studies [25, 37, 67]. We pre-filled sticky notes with common challenges, including individualizing goals or homework to client needs [25, 67], assessing client barriers [67], placing demands on clients [25], not knowing when/where/how to do homework [25], and lack of caregiver involvement [25]. The sticky notes were identical for both therapists and clients, but we changed the directionality of the phrasing. For example, we included the challenge: “lack of caregiver involvement” for therapists, and “other people in my life are not involved” for clients. The pre-filled sticky notes were intended to help participants see examples, use them as a starting point to generating other challenges they encountered, and reflect on which challenges were most relevant for them.

Before the interview, we asked participants to email us a list of the biggest challenges they encounter in working with homework, which we also included on the sticky notes. We also included blank sticky notes if participants wanted to add other challenges they had faced. To focus the interview on the biggest challenges that participants encountered, we asked participants to choose four sticky notes representing their challenges with goal-setting and homework. Participants discussed how they went about addressing the challenges of designing and engaging with homework, and what could help them better design homework. Therapists discussed how they work with clients on identifying and adapting homework. Clients discussed how they work with therapists to identify homework, as well as how they go about engaging in homework outside of therapy sessions, and what could help them in this process.

### 3.3 Data Analysis

We used a mix of inductive and deductive approaches to create a codebook based on which we further analyzed data and conceptualized themes [23]. To create the codebook, we identified some codes through a deductive approach because we wanted to know how participants addressed existing challenges that we were aware of from past literature. The deductive analysis of the data was driven by existing challenges identified in prior work [25, 37, 67] such as forgetfulness, avoidance, homework difficulty, and busy schedules. This led to defining codes such as “challenge - homework completion - avoidance”. Next, we identified codes through an inductive approach. A researcher wrote memos after conducting each interview, which informed the codes. A total of four researchers coded data. First, two researchers conducted open coding on one transcript independently and then discussed the codes and resolved disagreements. The researchers continued to discuss two coded transcripts at a time over multiple sessions of analysis. We used a line-by-line descriptive coding approach [100] for inductive analysis. Example codes that we conceptualized through inductive analysis were: client’s negative emotions as barriers to pursuing homework, understanding context to set reasonable goals, and resistance to engage with homework. We conceptualized other codes from the memo writing, usually as more abstract categories, such as: barriers to engaging with homework, out-of-session challenges, and information needs.

We revised the code book over multiple weeks, and one researcher applied the code book to all transcripts. Two other research assistants applied the code book to 11 transcripts to support agreement on the codes. The first author continued writing memos throughout the coding process, and used affinity diagramming for organizing the codes. The senior researcher on the team and the first author conducted a second cycle of coding the data as a team, primarily conducting pattern coding [100]. Throughout the coding, affinity diagramming, and memoing process the research team conceptualized themes in the data such as: adapting homework difficulty and personalizing goals to client values.

### 3.4 Researchers’ Positionality Statement

The authors recognize that they have a relative privilege in the perspectives they hold towards mental health: they are all in positions in which they have access to mental health services. We are aware of the stigma that other groups experience in discussing mental health, although we do not experience it in the same way. Our privileged position might have impacted the participants we recruited, who might come from a more privileged background than others. We all identify as women, which might have drawn more women to speak with us, although we tried to mitigate this in our recruitment, by specifically looking for men and gender-diverse individuals on online platforms. The authors represent the perspectives of both clients and providers. We believe it is important to take a human-centered approach that seeks to understand and design for the perspectives of clients and therapists, and their lived experiences. We recognize there are power imbalances between clients and therapists. Throughout our interviews and analysis, we sought to listen to and represent both perspectives as reflected in the data.

## 4 RESULTS

We identify three challenges that clients encountered in engaging in therapy homework: homework difficulty not matching the client’s skills and level of motivation, environmental and structural barriers in everyday life, and the discomfort clients felt when trying to engage with homework. We find that clients and therapists use techniques of reducing homework difficulty, finding alternative activities, and creating safe spaces to manage discomfort.

### Types of homework.

First, we contextualize our findings in the type of homework that participants engaged in. Table 2 and Table 3 in the Appendix have a non-exhaustive list of homework that participants brought up during the interviews. Therapists tried to understand multiple aspects of the client’s life at the beginning of the therapeutic process to break down the client’s goals into homework and make them more specific and relevant to client’s context and values (T1, T2, T4, T5, T8, T12, T13). This up-front work offered a starting point for the therapist to tailor their support for the client. By knowing personal and contextual information about clients, therapists could suggest homework that was adapted to the client’s needs and resources (T2, T12, C7), increase client awareness (T12), prioritize what clients wanted to work on therapy (T10, T12), support continued engagement with goals over time (T1, T4, T8). Homework included activities aimed at building physical health habits, managing social connections and relationships, and practicing cognitive activities and thought management. Homework aimed to help the client set new physical healthy habits (T9) involved physical activity (T6, C8), going outside (T3, C12), doing art therapy (C10), modification of eating habits (C13) or drinking habits (T11). To manage relationships, involved social activities such as talking with others (T11, T13, C12, C13). Homework involved practicing relationship skills such as having better or difficult conversations with others (C11, C13). Some homework involved activities to help the client achieve the goal of getting a job, such as writing a resume (T9). Other homework was focused on activities targeted at improving cognitive states such as meditation (T5, C6, C14), mindfulness (T5, C2, C7), breathing exercises (e.g. deep breathing) (T11, C7, C13), grounding exercises (e.g. describing things in the environment) (T11, C1, C14), or thought activities (e.g. restructuring thoughts, setting certain thoughts aside when it is not productive to think about them; visualizing a safe space) (C8). Participants used worksheets to practice skills toward their goals (T3, C9). Worksheets involved reframing thoughts, trauma understanding, distinguishing thoughts and feelings (C10), and writing down thought processes (C11). Journaling was used to write about thoughts and feelings (T4, C1, C2). Clients with anxiety sometimes practiced exposure, where clients can consistently approach what they are fearful of, such as touching something that is dirty when the client is afraid of getting sick from it (T8).

### 4.1 Tailoring homework difficulty.

Clients found it challenging to engage with homework when they did not feel motivated or homework did not match their skill. If the homework was too difficult, they might not know how to do the homework, or not have the skills to complete it, while if it was too easy they wanted more of a challenge. To tailor homework to client skills, clients and therapists adjusted homework difficulty by reducing magnitude, adjusting timing, duration and frequency of behavior, or adjusting the homework to the client’s knowledge.

#### 4.1.1 Challenges in pursuing homework that did not match client skill or motivation level.

Client engagement with homework varied based on their therapy skills level. Some clients with less therapy skills experience did not do the homework because they were unsure how to do it (T9, C1, C9, C10, C11). For example, C9 did not know where to start homework, so they delayed it: *“sometimes I don’t start, and I put it of for a little while”* (C9). Some clients (C1, C9, C10, C11) felt that the homework did not match their skills level, such as homework that required them to have coping skills for managing emotions when they are not sure how to do that yet (C1). This made the homework feel less helpful: *“I feel like sometimes the strategies given to me are not really helpful… I don’t know… how do I cope with this? … Or how far I can go with just dealing with the emotions?”* (C1).

Sometimes homework was not aligned with clients with more advanced therapy skills and experience, such as already knowing how to process emotions and not needing to journal (C7), or only being offered the same types of meditation (C6). They felt the homework was irrelevant (T13, C6, C7), or not challenging enough (C6, C7). For example, C7 had enough skills to understand about their trauma, and did not feel that some of the homework was necessary for them: *“These workbooks… it seems like some of these things are too elementary. They’re not challenging enough… And maybe this is because I’m not new to therapy”* (C7). T3 tried to provide information about the therapy process to a client when the client felt that the homework was too simple: *“‘this is so simple, I don’t really need someone to tell me to call my son at 6pm’, and part of the therapy is doing enough psychoeducation and really bringing them into the whole process”*.

Multiple participants found that low motivation came in the way of working on homework (T1, T3, T4, T9, C1), even when it involved small goals: *“when you talk with a patient in a session about setting goals, even if they’re tiny, little goals, that patient can really, really struggle to get motivated to do any of it. And that’s a huge barrier to completing homework that’s going to improve their mood and get them out of their depressed state”* (T1). Clients avoided engaging with homework due to low motivation: *“avoidance… there’s often apathy built into the symptom of depression or just kind of inertia that makes it really hard to, even if we have a plan set up… to follow through on that”* (T13).

#### 4.1.2 Adjusting magnitude of homework.

To scale down the magnitude of the goals or homework, therapists might reduce the difficulty of the behavior based on the domain. For example, for a client with social anxiety, reducing the magnitude of homework to make it easier might involve reducing the number of people they have to engage with: *“a goal could be like, go to a party… they come back the next week, and they’re like, I just couldn’t do it, it was too hard… we might need to set a smaller goal, like, go out to dinner with one person”* (T7).

##### Adjusting duration and timing of behavior.

To make the homework more achievable, clients and therapists decided to reduce time spent on a difficult behavior. This could support clients who felt they didn’t have enough time for homework or were overwhelmed with other problems: *“if you can’t go on a walk for 30 minutes every day, can you do 10 minutes?”* (T11). In some cases, therapists may recommend to clients to adjust the timing of the behavior, to prevent them from relapsing. For example, T4 explains how delaying a behavior is progress compared to lapsing right away: *“from like eating disorder treatment… rather than goals having to do with not purging after a meal… will set delay goals. So can you delay purging by a certain period of time?”*. Therapists also help clients to adapt the duration of homework to the minimum amount of time that the client has (T9).

##### Adjusting frequency of behavior.

Therapists encouraged the clients to change the frequency of a behavior that was difficult to change for the person, to make it easier for a client to make progress and not give up on their goal. This adjustment could occur in the moment, when a relapse is at risk of happening: *“Let’s say that a patient set a goal of abstaining from alcohol for the week… the second day after a therapy appointment, they had a drink… And they’re like… I’m just gonna drink all week long”* (T3). To help the client stay on track, therapists propose a compromise of performing negative behavior, but for a shorter period *“could we just like reduce your risk of harm for the week? Maybe you’re going to drink but could you drink a maximum of two drinks a day for the rest of the week?”* (T3).

##### Adjusting homework to client knowledge.

Clients were not always knowledgeable about how to complete the homework. They felt like they needed aids to engage in the homework, such as explanations, examples of how to complete the homework (C10), and of problem-solving strategies that are used in therapy (T9). That could support clients in completing homework at their current client’s skill set and experience: *“examples of how somebody else has filled it out, or might fill it out… and how to answer… rather than just ‘tell me about a time that you were upset,’ having more specific examples or prompts”* (C10). When the client had more therapy experience and advanced self-management skills, they wanted homework that would go beyond the basic skills and give them new insights and motivation: *“when I’ve been in the therapy session and they given… something that where I gained some kind of aha moment or the light-bulb went on, or a new perspective about something… that can create the motivation to be able to explore it a little bit more”* (C7). Another client wanted to have novel elements to the same activity, to make it more engaging (C6) such as new music to meditation practices.

### 4.2 Tailoring homework to surmount barriers

Fifteen of the participants mentioned events in their lives or their clients’ lives that created barriers to engaging with homework. Clients experienced external barriers, such as environmental and societal challenges, and other demands and priorities in the clients’ lives. We describe how therapists and clients identified homework alternatives to address barriers.

#### 4.2.1 External barriers to engaging with homework.

Clients encountered environmental and societal challenges that posed a major barrier to engaging with their homework (T5, T8, T9, T10, T12, C4, C6, C7, C9). Barriers that came in the way included: house chores (C3, C6), lack of support from loved ones (C3, C7, T4, T5, T10), relationships (C3, T10), work or school (C5, C13, T10), child care (C3, C4, C7, C12, T8, T9, T10), financial problems (C5, C6, C7, T2, T5, T8, T12), other health issues (C9, T4), maintaining a diet (C3).

Such challenges included a lack of access to basic needs (e.g., money to pay bills) (T5, C4, C6), and a lack of reliable access to resources (e.g., computer or internet access) (T9). Clients who did not have structural needs met had more difficulty in working towards their homework. For example, a client who was in the process of applying for jobs but did not have reliable access to a computer which made it difficult to create their CV: *“for them to get to a computer, it was taking a bus half an hour to the library”* (T9). Structural barriers and systemic issues are not easily solvable (T8, T9): *“that’s something that’s hard to manage outside of the session… we can’t give our clients $10,000 to pay of all their debt and… be able to take some time of work to better their well being”* (T9). Childcare was brought up as a challenge for clients who wanted to focus on homework: *“it’s hard to manage that when life, you know, you’re having three kids and having to focus on doing this stuf* [homework]*”* (T10).

When barriers came in the way it could lead to the client and therapists disengaging from homework to favor more pressing needs: *“when just their lives are super chaotic… I have a client who was teaching during COVID… that interfered with therapy… we just didn’t do homework for a period of time and then had a breakup..*. [with a partner] *didn’t do homework for a period of time* [after the breakup] *…Just outside life gets in the way of just being able to focus on the issues”* (T4). Times of transition also made it harder to prioritize homework: *“I moved to here and started school and in a matter of months, I feel like my mental health has sufered immensely for having started this program… I suddenly had all these demands on my time”* (C5).

Participants wished for support from their jobs and family so they could take time of to prioritize their mental health (C4, T9, T10). C4 did not feel supported from their job and felt punished when taking time of from work: *“there’s no support for really taking time of, to recharge, so that you can come back and be better.”* Participants wished childcare received more social support *“something more, or family support, more like coaches, more time, more childcare”* (T10) and financial support: *“childcare is really expensive, and cost prohibitive for a lot of people. So if there were some kind of formal social support that gave you 20 hours a month of certified paid childcare… to spend your childcare budget, however you need to, would give people an opportunity to just take some time away, or go through go to their therapy appointments”* (C4).

Due to having many demands on one’s life, finding time to engage with homework could be difficult (C5, C7, C8), stressful (C7), and overwhelming (T5): *“I’ve just got… lot of things to do and things to take care of… it’s just too much. So sometimes, it feels like I don’t have enough time. And it’s stressing to, to stop and oh, wait a minute, I’ll work on some mental health therapy issues”* (C7). During times of high demand, clients had a hard time finding any time for themselves, which made homework even harder to do: *“if you’re taking care of a young child and everyone else in your family is sick. Just finding those precious 5, 10 minutes to carve out even just a little bit of time for themselves”* (T9).

#### 4.2.2 Finding alternatives to surmount barriers.

Participants said that, often, the original homework set in therapy was no longer feasible between sessions due to barriers in their lives. In those cases, therapists thought it would be useful to have homework alternatives with a similar role to the original homework. Participants thought it was important to anticipate barriers the client could encounter and to have access to alternative homework to overcome barriers.

##### Alternatives with similar role.

As a therapy strategy, participants found it important to have alternatives to their intended plan when things come in the way (T2, T7, T11, T12, C1): *“what are my steps for overcoming* (a barrier)*? ‘What is my plan B?’ And I think sometimes using the phrase Plan B is really helpful because that’s a common expression that people understand”* (T12). The types of functions that homework might have depended on the client’s situation and could include: engaging in activities that improve mood, such as being more physically active, getting out of the house more, engaging in activities to understand and process their feelings, problems, or traumas, such as journaling or workbooks, avoiding or delaying an activity to stop a negative behavior, such as weighting oneself frequently or eating types of food that one has an intolerance to.

During therapy, therapists and clients identified another goal that satisfied the same role as the original even if it was a completely different activity (T2, T7, T11). To find an alternative, the activity might be something completely different, as long as it fulfilled a similar role: *“if they can’t go on a walk, can they dance for one song every day?… we want to find some sort of movement. I don’t care what kind of movement it is”* (T11). Another approach to finding alternatives was to identify what the client is willing to do that has a similar function: *“if they say, Oh, I can’t leave the house, but you’re willing to leave the house to go grocery shopping outside. Okay, so how can we work that into your grocery shopping trip?”* (T2). However, alternatives were not always offered, which led to discouragement: *“I was expecting maybe another strategy or another tool that I can like pick up, but it was like nothing else was offered”* (C1).

##### Alternatives in anticipation of barriers.

Therapists tried to anticipate with clients what could come in the way of the homework. That could support clients to problem solve when problems happened between sessions: *“For therapy in general, or also for home practice, what might get in the way, if they’ve got kids at home might… interrupt them… they need to attend to elderly parents that also live at home, they get so busy with work. And then we’ll try to kind of problem solving that ahead of time”* (T5). A therapist suggested a technology tool that could prompt the client throughout the week to work through internal and external barriers that came in the way of doing homework: *“send notifcations to the person’s phone at certain intervals throughout the week and ask like, have you been working towards meeting goal 123?…. And if the person says “no”, then there could be a ton of follow up questions about what’s getting in the way. And if it’s avoidance, here’s specific strategies you can use to kind of name that, notice it, be comfortable with it, and be willing to feel the anxiety anyway”* (T1).

##### Assistance in identifying alternatives.

A client wanted to receive alternatives to homework between sessions at the moment they experienced difficulty completing homework: *“if there was like something… that helps me in that situation. I could have backup options apart from journaling, this is what you can try”* (C1). C1 received such help during therapy sessions after the fact, but found it to be too late: *“this always comes up in the next therapy session. But it’s not really helpful. Because that moment when I needed that advice is probably gone”* (C1). A therapist imagined that technology could provide alternatives by including a list of activities: *“if we chose a goal that was not achievable that week… They could choose like that similar one, like without my input”* (T7).

When the client had more therapy experience, they started independently adapting the homework to their needs. Clients said that they would find other options of activities to substitute the homework (C1, C3, C4, C7): *“Sometimes it’s journaling* (that C7 avoids) *because I feel like I’m just repeating what I have in my head already. Why am I writing it down… sometimes I already talk this stuf through my head… And then I’ll say, ‘Well, okay, what do I want to do about it?’ Maybe I’ll go out and go into nature, go for a walk or go out and do some deep breathing or something like that. So then the journaling becomes like a moot point”* (C7).

Mental health barriers can make it difficult for an experienced client to adapt homework in times of distress, as C4 expressed: *“I really do have a good foundational knowledge. And I really do implement a lot of these strategies just in my life and with my children. But my issue was, when I would get in these emotional flashback moments, I had no access to cognitively what I know how to do. And so until I could get myself emotionally regulated, I couldn’t access any of the other things that I know.”* C4 said that homework could include resources to help the client in case mental health setbacks (e.g., “spiraling”, negative self-talk, and struggle to emotionally regulate) happened when the client tried to engage with homework: *“And so that’s why the homework was really specific about if this happens, here’s your toolbox, because that was what I asked for. And that was what I needed.”*

### 4.3 Tailoring homework to manage discomfort.

Clients often felt uncomfortable when doing homework, which led them to avoid engaging with it (C10, C8, C12, T1, T7, T8) due to anxiety and depression symptoms (T1, T3, T8). Clients and therapists adapted homework to manage discomfort and engage with homework such as creating recovery time after homework, de-escalating emotions, creating a safe space for doing homework, feeling as if they are far from their problems, resurfacing how the clients’ values connect with the homework, and providing encouraging feelings to the client when they felt overwhelmed.

#### 4.3.1 Homework activities were uncomfortable.

Therapists (T1, T2, T7, T8, T12) and clients (C5, C7, C8, C9, C12, C13) stated that clients with depression and anxiety often experienced discomfort as part of the mental health condition they are experiencing, in the form of intense emotions (C5, C8, T3), negative self-talk (C5) and physiological responses (C12): *“what we ask them to do is oftentimes uncomfortable”* (T8). While engaging with homework, clients often experienced moderate to intense emotions (C5, C8). They described the experience as feeling anxious (T3), uncomfortable (C12, T8), overwhelmed (C8, T9), ashamed (C10), and fearful (T1, T3, T8). In some cases, they also experienced physiological symptoms such as an elevated heart rate (C12), trouble breathing (C12), and urge to cry (C5). The discomfort was particularly pronounced for clients who were managing anxiety along with other disorders (T3) or had multiple intersecting barriers (T3, T5, C5): *“A lot of my clients when they start DBT their life is a mess. Many of them are not working or… regularly engaging in self-harm, multiple substance use problems, eating disordered behavior, lots of relationship difficulties… Life chaos plus lack of motivation, or like lack of belief they can change… So avoiding* (homework) *is a hallmark”* (T5).

Clients were reluctant to engage with the homework, and some avoided it altogether due to the feelings they would experience while working on it (C10, C8, C12, T1, T7, T8). For example, C10 did not want to experience vulnerability and shame: *“I just didn’t want to do it… being vulnerable or feeling ashamed or being honest about what I’m feeling… and sometimes saying it, doing it, makes it real. And I want to not… be real about something.”* C12 avoided homework due to uncomfortable physiological symptoms: *“even just talking about the problem… your heart rate goes up… cognitively, you’re all distorted… it’s uncomfortable to work on that thing* (homework) *“physically… oh my god, I’m sitting there uncomfortable”* (C12). For other clients, homework involved exposure to their source of fear or anxiety as part of the treatment: *“not wanting to do the scary things that bring on anxiety and like fearing the outcome, not knowing if they’ll be able to tolerate the anxiety that the situation might produce”* (T1).

Clients found it difficult to engage in behaviors that were unfamiliar, even when they wanted to implement change in their lives: *“You can know a habit is unhealthy but it can be hard to quit… So you can know that you need to make changes in your life, or that whatever you’re doing is amazing, like the right way, but it’s kind of hard to quit that because it’s unfamiliar territory that strays from that* (C14). C14 recognized that it was necessary to be uncomfortable sometimes during the process of behavior change, throughout the therapy sessions: *“Familiarity is kind of comfort thing, but it’s no longer serving you. Because over time you change. Familiarity is, at this point, unproductive, you know, it’s like as a negative influence on my life”* (C14).

##### Inappropriate timing for experiencing discomfort

Some client homework was trigger-dependent. For example, certain types of homework required the client to engage in a therapeutic activity when the client had negative thoughts: *“this specific exercise, you know, it’s trying to analyze some negative thoughts that I have. So when I have to recognize them, instead of moving on from it in some way, I have to engage with it”* (C5). This can be particularly difficult, because the client might be in a situation in which they are not able to face those negative thoughts, for example when they are in a social, public setting with other people: *“that can be quite difficult, especially. I don’t get to choose when that happens, you know, and most of the time, I’m in public with other people. And all of a sudden, I’m feeling some really intense emotion as a result of kind of noticing this thought”* (C5).

#### 4.3.2 Creating comfortable space and time for managing discomfort.

When clients were feeling overwhelmed, they wanted to find a safe space away from their problems, where they could de-escalate their emotions: *“I could just be in like a beautiful calm, quiet space. Somewhere more warm and lovely”* (C8). C12 imagined that Virtual Reality (VR) tools could help clients feel they are in a more comfortable space to work with homework: *“it could be really soothing to wear your 3D glasses or whatever, and go somewhere else, so to speak, and relax… it’s like you want to go somewhere else. And you just can’t”* (C12). C8 felt like they needed space away from their problems to feel better, in order to be able to address them: *“creating a little bit of physical distance between myself and the problems”*. Even when participants want to engage with homework, it took time to recover after finishing the homework due to the negative emotions that occurred while doing homework: *“I’m going to need that time afterward to really recenter… it’s that brings up uncomfortable feelings”* (C8).

#### 4.3.3 Preparing for challenging moments.

Therapists provided in-session support to help clients in managing future momentary distress related to homework. They did this by helping clients with proactively planning (T10), practicing homework in their presence (T8), and increasing client self-awareness about their thoughts (T10, T8). This helped clients with managing negative feelings when they occur in the moment: *“Coming up with more adaptive statements that they’re not able to access when they’re feeling really depressed and having that readily available. So when they’re feeling depressed, they can remind themselves, this depressive thought is not actually accurate. It’s just my depression talking to me”* (T8).

Clients encountered uncomfortable feelings during moments when it was inconvenient to stop and pause to do homework, such as when they were in the car driving (C4). They thought digital tools could support them in the moment, when they were feeling overwhelmed, to deescalate their negative emotions (T1, C4, C8, C12). Participants desired an easy way to journal in the moment (T1, C4): *“in those moments where… I have this intrusive thought that comes into my head, if there was some really easy to access, digital journal or recording journal where I could just kind of say, ‘oh, gosh, here’s what it is, here’s what I need to do’”*(C4).

#### 4.3.4 Surfacing homework benefits and client values to manage discomfort.

Therapists mentioned that surfacing homework benefits could support clients to engage with homework even when it was uncomfortable. Therapists reminded clients of their goals and values and how those connected to the long-term benefit of engaging with homework. Clients thought that encouragement from the therapist outside therapy sessions would be useful because they knew the client’s goals and values and why they needed to do homework.

##### Surfacing homework benefits.

Some therapists saw the discomfort of the homework as a temporary negative experience, that could lead to the clients feeling better in the long term. However, T3 found it difficult to show the long-term benefits to the client: *“patients don’t always see… this really is gonna pay of in the long term… but I can’t get them that long term payoff… that positive feeling that’s going to come down the line from reducing their symptoms early on”*. T3 sought to provide some form of visible short-term benefit to the client, even if the homework was uncomfortable by trying to remind clients of the value of homework in the long term. Clients thought it would be helpful to use technology to hear examples from others who benefited from engaging in homework: *“an alternate technology where it’s like… people giving their personal experiences: ‘okay, this is what I’ve tried. And this is what’s helpful. It’s better than hearing it from a person who’s tried and tested it..*. [compared to] *a set of activities recommended from like a textbook of the therapist”* (C1). Some therapists and clients also felt that surfacing homework benefits were also valuable when clients experienced low motivation. C2 felt more motivated when thinking about the benefits that homework had brought them: *“I don’t want to hit rock bottom… I think trying to acknowledge my progress [using the therapy tools], I think it’s something that kind of motivates me.”*

##### Connecting homework with client values.

When the clients were having a hard time engaging with homework, therapists used client values, and how they related to the client’s goal to provide encouragement (T1, T4, T8): *“Why are you doing these really hard things? There’s a reason why you’re choosing not to just continue on with the status quo. And you’re choosing to engage in these oftentimes really hard and uncomfortable therapies. I think tying it into those values, those things that you hold really strongly and are really important to you, can help you do the hard things that oftentimes kind of suck”* (T8). T3 thought technologies could monitor what skills clients practice, and send the client reminders to practice a skill by connecting that skill to a value: *“you know, doing this is going to help you meet that goal of going out to dinner with your friends”* (T3).

## 5 DISCUSSION

This study has investigated the practices of mental health therapists and clients in setting and implementing therapy homework, and their needs in engaging with homework. To support client engagement, we found that clients and therapists made a range of adaptations to therapy homework. Homework needed to be adapted to the client’s skill in terms of difficulty and resources needed to complete homework activities. When clients encountered barriers, clients and therapists adapted homework with different types of alternatives. Because homework was uncomfortable to engage in, it could require extra preparation and a safe environment.

The lessons learned from clients and therapists tailoring homework give us insights into how technologies could better support the tailoring of digital mental health interventions, such as motivational messages and recommendations for mental health activities. Intelligent systems are increasingly being used to support mental health and provide personalized experiences. Conversational agents provide CBT interventions [43] or emphatic responses to people in need [81]. Just-in-time interventions [20] deliver different text messages, or tailor activities based on the user’s context [20], medium (e.g., video vs text), length of content, or visual display [14]. Mental health therapists have been recommending the use of commercially available mental health technologies to their clients [106]. The types of homework activities we studied are part of numerous mental health self-management technologies [79]. Through our findings, we have expanded the understanding of design considerations for tailoring mental health technologies by drawing on the expert practices of therapists, and client’s and therapist’s lived experiences of tailoring therapy homework. Our findings inform how different variables might be used to adapt interventions (e.g., location, timing, expertise, and motivation), and how intervention difficulty and options might be adapted. We discuss how mental health technologies that offer activity recommendations can better support people using them, and areas that future work can investigate in tailoring for mental health. We further discuss limitations of tailoring technology to support client barriers, and considerations for engaging with systemic barriers.

### 5.1 Tailoring variables: How contextual information can support adjusting digital mental health interventions

Tailoring variables are information about the individual, used for customizing a digital intervention to decide when and how to intervene [84]. They enable adaptive interventions to different situations that a person might be experiencing. We found that therapists used varying criteria to tailor homework activities to client needs. We discuss how we can leverage variables such as location, timing, skills, and motivation to adjust homework activities through technology.

#### 5.1.1 Adapting digital interventions based on the individual’s expertise level.

Many psychosocial treatments (CBT, DBT, ACT, etc) rely on the client learning and applying skills to manage their mental health. such skills are also reflected in technologies that support people in therapy-related activities (e.g., setting goals, journaling, identifying barriers, and cognitive restructuring) [79, 104]. Prior work shows that users find it useful to choose from activities that allow them to practice different skills when using mental health apps [104]. Our work surfaces that client engagement with homework or mental health activities depended on skills acquired through experience and practice (section 4.1.1). When clients did not have enough instructions, low skill levels reduced the likelihood of whether they did an activity (e.g., a less experienced person may not know how to de-escalate emotions when feeling overwhelmed, while a more skilled person could use techniques learned in therapy to feel less overwhelmed). The skill level related to mental health activities that people have for completing different types of activities varied and determined which activities clients were willing to engage with.

Based on our findings, mental health technologies could adjust the recommended therapeutic activities to the skill of the user. If the user is a beginner, they might need more information and structure around how to do the activities and why. As the user gets more experience, such information is less necessary. Technologies could include onboarding experiences where the client skills are assessed based on prior experiences [9], and reflections on what they were able to achieve in their past. Based on usage data and practice with different therapeutic activities, technology could encode a user’s expertise level with different skills, and update the activities suggested to the user as they gain more expertise. Assessing skills in the context of mental health activities can be particularly difficult because behavioral activities might span many domains, such as mental health, therapeutic skills, exercise management, diet management, and social situations. A client might be well-skilled at managing exercise, but not social situations. More work is needed to understand how to represent and capture client skills in a wide range of situations.

#### 5.1.2 Adapting interventions based on location and social context: (in)appropriate timing.

Past research suggests that location can be a useful dimension for tailoring mental health interventions [17, 88, 120]. For example, locations such as home, [120], building [17], or other movement [48] can be used to adapt interventions. Moments after social interactions could be effective moments for recommending interventions such as mindfulness [48]. We surface that some of the important aspects of location are how public a location is and the presence of other people.

The location and sociality of a situation influenced whether users wanted to engage in a particular homework activity, particularly when the homework was *trigger-dependent* (to be completed when a client faced a specific situation). For example, clients may have encountered a situation in a public space where they experienced negative feelings. The homework set during therapy sometimes recommended pausing and doing a therapeutic activity, such as tracking, acknowledging thoughts, and responding to the situation. However, that was not desirable in a public space. Apps that could sense the presence of others through Bluetooth activity [48] could maintain the privacy of the individual by subtly recommending engaging in an unobtrusive activity such as taking a deep breath. Because individuals might be uncomfortable with others knowing that they are engaging in a mental health activity, apps could have a display mode that maintains user privacy, in a way similar to how menstrual tracker research has proposed doing [42]. Alternative forms of journaling or representing experiences could be used when the user is in a public space, for example by using imagery to characterize their experience instead of only text journaling [90] (e.g., choosing photos according to an emotion). We found that other inappropriate timing issues occurred when a person was driving and could not pause to engage in a journaling activity. Technologies could help people use different modalities to engage in therapy homework. For example, if the homework involved journaling after a particular event or trigger, during movement time, apps could implement similar features to “driving focus” [1] to adjust the recommended intervention. If the user desired to engage in a reflection moment, an app could include a recording feature to capture information related to the homework.

For some clients, social situations could trigger negative emotions, which constitute times when a client should engage in homework activities. Such situations could be inappropriate moments to engage with homework, because of the client being in a social setting and experiencing intense emotions. Technologies delivering mental health interventions could be designed so that the user can specify triggers. Apps could recognize which contexts could be triggering (e.g., being at a party would be triggering for someone with social anxiety) and suggest a trigger-based homework. However, previous research suggests that technology could balance automated interventions and pre-scheduled activities since both are effective and support the clients’ decision about when to engage in behavioral activities [56]. With technology support, clients could have the flexibility to complete homework at a pre-scheduled time or at a trigger-dependent moment. For example, a client and therapist might agree that the client would start a conversation with someone in the next week. They could define a location (at a cafe) and a time. If the client used an app to keep track of the homework, and had not yet marked homework as completed, the technology could provide a nudge to engage in the homework when certain social settings are detected (e.g., when they are in the cafe).

#### 5.1.3 Adapting support to client low motivation and discomfort.

An opportune timing for supporting the client was when they had low motivation or were experiencing discomfort (section 4.3). Current apps regularly assess the user’s state and feelings (e.g., BloomCBT app), but they require self-reports. There is an opportunity to understand if and how motivation levels could be automatically detected for purposes of making recommendations. Such information can be used to assess if the individual feels discouraged and encourage them to engage in homework activities. Such mood assessment and response could be achieved through motivational interviewing—a therapy strategy that helps surface client’s motivations and encourages clients to commit and engage with their goal [77]. When a tool recognizes the client is not motivated (e.g., noticing the client is avoiding homework), it could ask the client to complete a motivational interview assessment to help them remember why they wanted to work toward that goal. Our findings also showed other strategies that can be incorporated into technology when individuals feel disengaged from working towards their goals. For example, resurfacing the long-term benefits of the goal clients committed to working on helped them feel more comfortable working towards that goal.

### 5.2 Intervention options: Adapting mental health activities to user’s needs

Intervention options are possible actions or activities that might be used in a technology design to decide which intervention to deliver at any given decision point [84]. Digital technology interventions can include support (e.g. information, feedback), can have different intensities, and can be delivered through various modalities (e.g. phone, app) [84]. Based on our findings from clients’ and therapists’ practices and their stated needs, we identified approaches for adapting therapy homework between therapy sessions. Approaches particularly targeted the intensity of the intervention (difficulty of activities) and identified interventions when users encountered barriers. We proposed design implications for how technology can support clients as they engage in therapy activities between sessions, specifically related to changing the difficulty of homework, providing alternatives, and offering relevant informational content to motivate clients. Because homework activities are typical therapeutic activities that might be part of apps, such design changes can improve mental health tools by incorporating therapeutic activities for self-management or providing recommendations for mental health activities.

#### 5.2.1 Adapting the difficulty of therapy activities.

We found that changing the difficulty of homework could be achieved by changing the duration, frequency, or how long participants engaged in positive behaviors (section 4.1.2). To support such homework adaptations, technologies could be adjustable. That is, self-management apps that include mental health activities (e.g. meditation, thought restructuring) could have options the user select to “make activity easier” or “make activity more challenging.” Conversational agents could respond to user responses such as “this feels too hard.” While modifications can be useful, clients might need guidance on how much they should change a particular behavior. For example, technologies could have default options for ways to make activities easier. If a client is just starting with an activity, like starting to walk more or getting out of bed, then any small change might be sufficient to move them toward their goal. Such time-based adjustments could be computationally incorporated into technologies that support activity planning by keeping track of activity progress over time. For example, after completing an activity, a client can rate it as easy or difficult. If one activity involved walking for 10 minutes and the client decided they wanted to be more challenged, the next activity could increase the difficulty by suggesting a longer walk (i.e., changing duration) or taking more than one walk a day (i.e., changing frequency). Suggestions could also adapt to other client commitments or locations [92]. In social situations, “reducing magnitude” could simply mean reducing the number of people that one interacts with. However, reducing the difficulty of the homework can be hard to quantify in other situations, and clients might need further support.

#### 5.2.2 Modifying activities by surfacing user values.

We found that adjusting homework could be accomplished by including relevant information about values when clients felt disengaged or unmotivated (section 4.3.4). This approach could help inform motivational messages. Capturing user values can be difficult to achieve even in a face-to-face setting by clients and therapists, and might be even more difficult to achieve automatically. To construct motivational messages, therapists and clients can use tools that can make it easy to communicate about values [19] and to track values about the therapy homework [7]. The resulting motivational messages tailored to client’s values could help clients engage with their homework.

#### 5.2.3 Identifying alternative activities.

Alternatives were important for supporting the client when barriers came in the way (section 4.2.2). Alternatives can serve as another approach for adjusting recommendations for mental health activities in digital tools. Alternatives had a similar functional role as the original activity. For example, an activity through which the client achieved physical movement could be done through exercise or dancing. It is important to understand the role that the behavior fulfilled to identify a suitable alternative. For a computational system to support identifying alternatives, we encourage developing encoding for the common functions that behaviors might satisfy for mental health and categorizing activities. In other domains, like physical activity, goals can be represented through encoding physical activities based on cardio, strength, or calories [11]. For diet, alternatives might involve suggesting foods with similar nutritional value or ingredients that provide a similar type of spice to other foods [33]. A client could also access a list of options to the original activity they planned to do. In addition, for activities that can change based on location, such as physical activity or places to eat, technology could use location to adjust recommendations for activities. Such alternatives are not always easy to quantify, therefore, more work is needed to understand if an approach that involves human support is needed.

### 5.3 Adjusting context to support engagement in discomfort of mental health activities

In addition to tailoring interventions according to user context, we also found a need to adjust the context of the participants to support their engagement with homework activities. Mental health activities that participants needed to engage with made them uncomfortable, which led to them avoiding the activities (section 4.3.1). This created tension for the therapists who wanted to support clients, and for clients who wished to engage in uncomfortable activities. Participants with more therapy experience in our study found that consistency in doing the homework and practicing skills helped them successfully change their behavior, even when those activities were uncomfortable and results were not immediately achieved. Pursuing uncomfortable activities was beneficial for the client to acquire skills, a finding consistent with prior research. Acquiring new behavioral skills happens through physical (e.g., feeling hot, hungry) or mental (e.g., psychological stresses) discomfort [101]. Prior work has not offered many solutions on how to design for feelings of discomfort, but there have been calls to be intentional about designing for uncomfortable experiences rather than allowing them to be accidental side effects [18, 101]. Our findings illustrate opportunities for using the techniques used by both clients and therapists to support people experiencing discomfort.

#### 5.3.1 Adjusting temporal engagement to support discomfort.

Prior discussions in HCI suggest that discomfort is associated with new activities [101, 121] and that recovery is important [101]. In our study, participants indicated they needed time after engaging in uncomfortable homework to recover (section 4.3.2). This finding can be extended to how people might be using mental health technologies for therapeutic activities. If activities pose feelings of discomfort to users, our findings suggest a need to anticipate recovery time for the individual to adapt to the new experience and recover from the negative emotions [101]. If mental health homework activities are designed as part of technology, the recovery time can be built into the activity and appropriately inform the user that the activity might take longer to engage in. Another option could include approaches from “slow technology,” which allows a longer time for interactions as a way to enhance the individual’s ability to engage with uncomfortable feelings [53]. Slow technology draws from “slow design,” which advocates for slowing down the pace of life and increasing the mindful use of products [52]. Slow technologies could induce deeper reflection about one’s own actions and have a positive impact on well-being [52].

#### 5.3.2 Adjusting the environment to support client engagement.

Participants in our study mentioned that when they felt overwhelmed, they desired a safe and comfortable space to de-escalate emotions and work on their homework (section 4.3.2). Technology has the potential to create digital spaces that feel comfortable for clients to work on therapy homework and have the potential to create new therapy experiences. Some participants mentioned that virtual reality could be used to take them to a place that felt more comfortable. Designing virtual/augmented/mixed reality environments for mental health treatment could help people reduce anxiety and depression symptoms [86], and de-escalate negative thoughts [51]. Other approaches to creating comfortable spaces include showing clients representations of digital gardens, which can reduce stress and depression [110, 113]. Japanese gardens in a digital application have relaxing aesthetics and could motivate users to engage in tasks [38]. Music could help clients feel more relaxed before doing therapy homework [118]. Online spiritual communities are yet another means to create a “safe space” and avoid negativity [109]. For example, in communities such as CaringBridge, people create a safe space through a community that shares spiritual beliefs [109]. However, similar to “time,” an actual safe and comfortable environment is a privilege and may not be accessible to all clients. Often, therapists’ work involves collaborative problem-solving with clients on how to create a safe environment to engage with homework.

### 5.4 Challenges and limitations in tailoring support for engagement in mental health activities

#### 5.4.1 Challenges of addressing structural barriers through technology.

Our findings show that sometimes people experienced structural and external barriers that were hard to address through homework activities, or the homework activities involved structural barriers outside the control of the individual. Structural barriers raise challenges about how mental health technologies can broadly support people struggling to surmount systemic barriers, and how we can achieve systemic change to support engagement in mental health activities.

In our findings, therapists and clients discussed how systemic barriers—including financial problems, lack of resources, and lack of childcare—can prevent them from engaging in mental health behaviors. These barriers are known to create disparities in access to mental health [36]. Some participants desired policy changes, such as having more access to childcare or time of work, that would make it possible for clients to engage in therapeutic activities. However, these changes need to come at a policy level and are hard to achieve through technology, so we must attend instead to research that shows technology has the potential to address disparities in mental health by increasing access to evidence-based treatments and interventions [95]. We recommend carefully considering how technologies are designed for people who might be experiencing systemic barriers. For example, racial and ethnic minorities are less likely to access mental health treatment but might benefit from digital interventions [95]. An important way to design for minoritized groups is to culturally tailor technology to address issues such as stigma [95]. While our work shows criteria for tailoring interventions, more work is needed to understand how cultural tailoring should be designed for engagement with therapy activities. Our participants also raised the issue of caregiving as a barrier to engaging in therapy homework. Since care duties are disproportionally performed by women [74], caregiving barriers highlight a need to better understand how technology might be able to support women’s engagement in mental health activities.

In our study, we have made recommendations about tailoring. For example, we suggested that recovery time can help alleviate the discomfort of therapy activities. However, it is important to highlight that access to time is a privilege. Often, disadvantaged populations have competing priorities that make focusing on health-related activities unsustainable [124]. Adding extra time to mental health activities might not be feasible for some people and could lead to inequities by amplifying who has time to access mental health resources. For those seeing a therapist, this suggests the need for some clients to practice homework during the therapy session instead of between therapy sessions. Alternatively, technologies have emphasized engaging in micro-task activities during moments that might not be otherwise used [115]. For example, technologies have been used to support people learning a language while waiting for an elevator or waiting in line [28, 29]. Physical activity is another consideration when designing technology with an awareness of systemic barriers. For example, if an individual’s neighborhood is unsafe, a system can help identify spaces where people feel safe and recommend them for physical activity [99]. If an individual does not have access to the gym, a system can recommend activities they can do without equipment [9]. More research is needed to understand what are appropriate alternatives to activities when people are faced with systemic barriers.

#### 5.4.2 Challenge of tailoring mental health technologies to user’s context.

Supporting successful tailoring as part of technology design is difficult to achieve, particularly given the added challenges of mental health. Successful tailoring based on context relies on accurate sensing and predictions of the user’s context. However, sensing or reporting context can be difficult. Tailoring variables like motivation or discomfort can be difficult to sense and require self-reports, but constant user self-reporting for adaptive interventions can also be burdensome [15]. Intelligent systems might not include diverse datasets that include information for their training data about places individuals consider to be safe locations to engage in activities [99]. Certain recommendations can worsen mental health. For example, in a mental health study, some participants reported their well-being worsened due to the chatbot’s mistakes and long response times [91]. Intelligent systems need to be sensitive to the mental health conditions that people might have. For example, making a dieting recommendation to a person with an eating disorder can trigger harmful behaviors [78]. Making recommendations that are not a good fit with the needs of a person with anxiety or depression, might make them feel like they are failing at their goals, exacerbating their negative symptoms. Sharing information through context-aware systems has been a longstanding privacy concern of users [16]. Research in context-aware technologies for mental health suggests that only necessary client data should be stored in databases[49]. Technology should avoid unnecessary client data exchange or external storage and be transparent to users about how data is collected and managed [49]. Future work can investigate how the design of mental health recommendation systems can incorporate a wide range of contexts using the “bittersweet content” approach [76], which proposes a way for engaging users in designing recommendation systems for sensitive experiences. Designing successful tailored systems, which re-emphasizes the importance of working with a therapist to receive tailored interventions at the moment, might still be a far-of target.

### 5.5 Considerations for tailoring to different types of homework

Participants in this paper used therapy homework as part of a variety of therapy approaches: CBT, DBT, ACT, CPT, PST, BA, and integrative therapy. Although these are different treatments, some homework, such as engaging in behaviors and cognitive reframing, are common across them. Participants mentioned several types of homework and exemplified their challenges with them. Therefore, our findings about adapting homework extrapolate across different types of homework, identifying a wider range of tailoring approaches than if we were looking at only one type of homework. However, a limitation is that some of the tailoring strategies we identified might not apply to all homework activities. For example, it might not be possible to alleviate the difficulty of a cognitive re-framing activity by shortening its duration. Further work is needed to implement and evaluate the types of adaptations we identified in this paper for different therapy approaches and different types of homework. For certain types of homework, for example, activities that traditionally involve paper worksheets would need to be redesigned for a digital format and more research might be needed to understand how to account for adaptation to difficulty of activities or to find alternatives.

## 6 LIMITATIONS AND FUTURE WORK

All therapist participants had at least 3 years of experience practicing therapy, and we only interviewed one therapist with more than 10 years of experience. Therapists’ lack of experience could have influenced our results. Our study therefore illustrates approaches taken and challenges encountered by *younger* therapists when designing therapy homework. Among clients we interviewed, there was an imbalance between the number of self-identified women (11) and men (3). Our gender ratio is more imbalanced than that of women to men going to therapy in the general population, and it leans in the same direction. Further work is therefore needed to capture the experiences of a more gender-diverse population.

## 7 CONCLUSION

In this study, we interviewed therapists and clients about their experiences with mental health therapy activities. We identified challenges that clients experienced in engaging in therapy homework, including not knowing how to start, having low motivation, experiencing discomfort with homework activities, and facing external barriers. We also have identified adaptations that clients and therapists made to therapy homework such as reducing homework difficulty, creating a safe environment, making barrier-driven adjustments, and identifying alternatives. Technology can support clients engaging in mental health activities through support at opportune moments, tailoring activities based on the client’s experience with therapy and motivation in the moment and context. When designing for mental health needs, technology could alleviate discomfort by adding recovery time after the client’s engagement with mental health activities or by supporting the client in remembering the long-term benefits of mental health activities.

## Figures and Tables

**Table 1.** Challenges for clients to engage with therapy homework between sessions and approaches to tailor homework in response to challenges

Challenges in engaging with homework	Approaches to tailoring homework
Homework difficulty Client lacked skill or knowledge Client did not feel challenged Client lacked motivation	Adjust magnitude of homework Reduce duration, timing, frequency of behavior Adjust homework to client knowledge Examples, activities that provide a challenge and engagement
External barriers Chores, lack of support, work, caregiving, finances, health Basic needs Emerging priorities	Alternatives with a similar role, backup plan Alternative in anticipation of barriers Resources for how to address barriers
Homework is uncomfortable Feelings of vulnerability, shame, physiological negative feelings, fear, anxiety Unfamiliar activities Timing of homework might not be conducive to engagement	Creating comfortable space Preparing for challenging moments Surfacing homework benefits and connecting with client values

## CCS CONCEPTS

- **Human-centered computing** → **Empirical studies in HCI**; *Empirical studies in collaborative and social computing*; *Collaborative and social computing theory, concepts and paradigms*; • **Applied computing** → **Health informatics**; **Consumer health**.

## A PARTICIPANT BACKGROUND

**Table 2.** Therapists (T) participants’ demographic information and homework examples they brought up during the interview

ID	Age	Gender	Race	Homework Examples
T1	26	Woman	Middle Eastern	Cognitive strategies: name what they are feeling (e.g., avoidance), be comfortable with feeling it; reflect after homework/ what client has learned; have clients try to eat a bit more (for eating disorders)
T2	28	Woman	Caucasian/ White	Behavior chart; exposure (for anxiety) to put the client in a situation that is uncomfortable
T3	36	Woman	Caucasian/ White	Worksheets (e.g., emotional arc) or apps to log sleep or track mood; mindfulness/ meditation; exposure: getting a client, who doesn’t want to leave the house, to walk their dog once a day, or take the dog to the backyard; abstaining from alcohol for a week (for substance addiction)
T4	39	Woman	Caucasian/ White	Exposure: eating fear foods, mirror exposure, delaying purging (for eating disorders); meditation or journaling (for regular anxiety)
T5	33	Did not identify	Caucasian/ White	Exposure: not washing hands, touching toilet seat (for obsessive-compulsive disorder); give a presentation (for social anxiety); behavioral activation: get out of bed and do something that day; short term homework: tracking mood, tracking avoidance levels; self-monitoring or DBT’s diary cards; mindfulness/ meditation
T6	29	Woman	Caucasian/ White	Running 1 mile 3x/week; eating ice cream 3x/week; meditation; going out with friends (for social anxiety); cognitive strategies: challenging a thought/ generating alternate thoughts, noticing thoughts/ distancing from thoughts
T7	29	Woman	Caucasian/ White	Changing behavior to change thoughts; exposure: go to a party or go out to dinner with one person (for social anxiety), diet self-monitoring (for eating disorders); logging thoughts (in a worksheet or digital document)
T8	31	Woman	Caucasian/ White	Reading and listening on 1st session: psychoeducation; podcast; exposure: touching toilet seat or watching someone getting their blood drawn (for anxiety); adaptive statements: reflection/writing down what the client has learned after exposure
T9	27	Man	Caucasian/ White	Behavioral activation (doing activities that are pleasurable and enjoyable); doing specific routines such as physical exercise 3x/week; write a resume (goal: apply for jobs); exposures
T10	32	Woman	Caucasian/ White	CBT techniques: managing intrusive thoughts (in worksheet or digital document); habit tracker app
T10	32	Woman	Caucasian/ White	CBT techniques: managing intrusive thoughts (in worksheet or digital document); habit tracker app
T11	33	Woman	Caucasian/ White	Behavioral activation: one self care activity per day (e.g., taking a shower, going for a walk 10 min/day), diary card once a day: writing the feelings you had that day; cutting down to 2 drinks/night; putting cold ice on face when feeling overwhelmed; connecting with friends
T12	53	Did not identify	Caucasian/ White	Healthier eating habits next week; how to do something else instead of smoking
T13	34	Did not identify	Caucasian/ White	What day and time is the client going to send an email to a friend they want to get back in touch; set appointment with financial planner; worksheets

**Table 3.** Clients (C) participants’ demographic information and homework examples they brought up during the interview

ID	Age	Gender	Race	Homework Examples
C1	26	Woman	Asian	Grounding: making a coffee or taking a shower in the morning to start a routine; journaling or texting themselves
C2	27	Woman	Asian	Journaling: “how can I be more mindful of my emotions this week?”
C3	43	Woman	Caucasian/ White	Cognitive reframing: sticky notes from takeaways from the session to refer back to during the week; avoiding to dwell on negative thoughts; journaling; podcasts; dates alone with husband; regular exercise; improving nutrition
C3	43	Woman	Caucasian/ White	Cognitive reframing: sticky notes from takeaways from the session to refer back to during the week; avoiding to dwell on negative thoughts; journaling; podcasts; dates alone with husband; regular exercise; improving nutrition
C4	53	Woman	Caucasian/ White	Pilates: it involves mindfulness and breath control; journaling: things they read and want to reflect later on, or identifying own thoughts; mindfulness app
C5	33	Woman	Caucasian/ White	Worksheets: tracking thoughts, how strong they are, and how they react to them; recognizing negative thoughts/self talk; breathing exercises; takeaway from the session to think about
C6	48	Woman	Asian	Meditation (app that therapist recommended); daily mental health assessment; cognitive reframing: think about what makes them happy
C7	62	Woman	Caucasian/ White	Journaling; deep breathing; mindfulness; cognitive activities: “how am I responding to a trigger? can I step back?”; workbooks earlier in therapy (to understand a concept)
C8	35	Woman	Caucasian/ White	Cognitive exercises: challenging and questioning thought patterns; mindfulness; putting certain thoughts and feelings into a “container” and setting them aside during times they are not helpful; centering activities: visualizing a safe space; more proactive activities: setting new habits/ routine, like physical exercise; worksheets (“prompts and questions”); writing a letter or journaling)
C9	41	Woman	Caucasian/ White	Cognitive reframing: journaling; worksheet: put down the thought, and reframe it more positively; dig in to see what is rational; reasons why the thought might be coming up)
C10	36	Woman	Caucasian/ White	Cognitive reframing: if facing a problem, what I tell somebody else how to deal with that?; journaling; art therapy; physical activity/movement)
C11	32	Woman	Asian	Improving relationship with family member: think of and write down dialogue or communication that would work well (defining topics of conversation), how to react or not when there is tension; reflect on own behavior to improve on it next time)
C12	40	Man	Caucasian/ White	Cognitive reframing: CBT exercises, call someone if needs social support, go outside and walk around)
C13	40	Man	South Asian	Improving relationship with family member: think how to have a certain conversation; breathing exercise; practical homework: rethink diet/ planning when to eat snacks and what type of snacks)
C14	27	Man	Caucasian/ White	Grounding: describe things in your environment; mindfulness/ meditation)
